# A Dictionary of Medical Science, &c.

**Published:** 1849-01

**Authors:** 


### ARTICLE VII.

*A Dictionary of Medical Science, containing a concise explanation of the various subjects and terms; with the French and other synonymes, notices of climate, and of celebrated mineral waters, formulæ for various official and empirical preparations, etc.* By ROBLEY DUNGLISON, M. D., Professor of the Institute of Medicine in Jefferson Medical College, Philadelphia. Seventh edition carefully revised, and greatly enlarged. pp. 912. Philadelphia, Lea & Blanchard. (From the publishers, and for sale by Keene & Bro., Chicago.)

A standard work so well known as Dunglison's Medical Dictionary, and admitted by all good judges, both in this country and in Europe, to be equal, and in many respects superior, to any other work of the kind yet published, needs no commendation from us.

The new edition, now before us, has been carefully revised by the author, so as to comprise all the new and interesting medical information acquired since the publication of the last. The mechanical execution of the work is equal to that of the best of Lea & Blanchard's publications. H.

